# Concept design overview: a question of choices and compromise

**DOI:** 10.1098/rsta.2023.0414

**Published:** 2024-10-09

**Authors:** Chris Waldon, Stuart I. Muldrew, Jonathan Keep, Roel Verhoeven, Terry Thompson, Mark Kisbey-Ascott

**Affiliations:** ^1^ United Kingdom Atomic Energy Authority, Culham Campus, Abingdon, Oxfordshire OX14 3DB, UK

**Keywords:** fusion, spherical tokamak, concept, powerplant, first-of-a-kind, design

## Abstract

The Spherical Tokamak for Energy Production (STEP) programme hypothesizes that a compact machine offers a route to reduced capital cost that directly tackles the barrier to entry of this potentially transformative technology. History has shown that with an unsolved, complex and highly interdependent design challenge, there is a need to balance exploration of the problem with progress. Almost all complex systems arise from the evolutionary improvement of simpler systems which is an approach the programme has adopted by working through a virtual natural selection of design families towards a single concept consistent with the initiating hypothesis. Issues are uncovered and solved more rapidly this way because the effort is focused on an end. In this current phase, STEP has had to be an agile fast-moving programme to work with what emerges as well as what was planned, to sit with uncertainty and to embrace self-organizing principles. The complex decision-making and compromises in emerging trades have led to a concept respectful of the tight aspect ratio hypothesis which carefully balances cost, performance and deliverability. It remains a high-risk and high-reward programme, but the character of the challenge is better understood building confidence and enhancing capability to advance the evolving design further.

This article is part of the theme issue ‘Delivering Fusion Energy – The Spherical Tokamak for Energy Production (STEP)’.

## 1. Positioning of STEP and learning the lessons from history

The Russian physicist Lev Artsimovich is purported to have said, ‘Fusion will be ready when society needs it’ [1]. Artsimovich’s assertion encapsulates a poignant reflection on the barriers to progress in fusion research, highlighting the pervasive influence of doubt and the elusive pursuit of perfection as formidable challenges that must be navigated before the realization of fusion aligns with societal needs. Societal need driven by the worldwide oil crisis of 1973 gave rise to a motivation to seek alternative forms of energy and financial support for the Joint European Torus (JET) programme researching whether fusion could produce significant quantities of power. This extrapolation from the devices then to JET’s scale was a big leap of faith that was accepted at a time when the appetite for experimental risk bearing was higher. This international sentiment saw Europe scale up JET from Tokamak de Fontenay-aux-Roses (TFR), the US transition from Princeton Large Torus (PLT) to Tokamak Fusion Test Reactor (TFTR), and Japan evolve from Jaeri Fusion Torus (JFT-2) to Japan Torus 60 (JT-60) with operations starting September 1984, December 1982 and April 1985, respectively. Even early in JET’s exploitation Paul Henri Rebut, JET’s principal designer, was conceiving the pathway to power generation with a bold three-machine vision [2]. His optimized programme proposed three devices to address long burn ignition (P1), concept optimization (P2) and materials testing (P3). P1 was the genesis of ITER but the initial cost estimates proved a significant barrier progressing this vision requiring a major redesign aiming to halve the cost [3]. Similarly, Rickover noted at the dawn of the fission power generation the appearance of unresolved conflict at every level and was mindful of the distinction between academic and practical approaches. In a conceptual phase, it is easy to change the design, lured by flawed optimization, without ensuring the shortcomings are uncovered. Adopting a simplified approach is prone inevitably to downstream recalcitrant conflicts, demanding substantial resources, time and financial investment for resolution through decisive action and compromise. Cost, complexity, development, rework and schedule were cited as practical attributes of Rickover’s pioneering programme delivering an unprecedented nuclear powerplant [4]. A large part of a nuclear plant cost model is associated with active facilities, and a reduction in size can, on the surface, seem like a tantalizing financial efficiency. The Jules Horowitz reactor made such a choice while the housed plant design was at a low maturity. This decision imposed a substantial constraint on the programme’s deliverability, resulting in a commensurate delay that posed a threat to the entire programme, prompting the need for strategic transformation and the implementation of a remedial action plan [5]. Nonetheless, cost remains an omnipresent impediment to entry for new power generation technologies given mature alternates are more established and readily deployable [6]. Consequently, a cardinal tenet of the STEP programme is designed for cost but noting Rickover’s implicit learnings of a pioneering programme: to drive progress by working through the conflicts and compromises.

## 2. Hypothesis and ambition

It is a starting hypothesis that a spherical tokamak (ST) is a reduced-size machine, compared with conventional counterparts, and therefore a reduced-cost machine. The capital cost of a fusion reactor is presently found to be dominated by the cost of the magnets used to confine the fuel, and the buildings that house the reactor [7]. In 1990, the UK pioneered the first operational spherical tokamak (called START). An ST confines fuel with much higher efficiency, driving achievable pressure up and capital cost down. In the case of START, UK scientists demonstrated a gain factor of four over conventional tokamaks [8]. Navigating this integrated challenge is complex, lacking definitive right or wrong answers, but involves weighing various pros and cons. An ST offers advantages in optimizing *Q*_engineering_, the ratio of the electrical power produced to the electrical power consumed by the fusion powerplant, by maximizing *P*_fus_ and bootstrap current fraction in a reduced radial build device at relatively low toroidal field by allowing high normalized pressure (*β*_N_) and high elongation (*κ*). The ST can operate continuously, a consequence of higher beta, eliminating the need to regulate electrical output. Architecturally, the open structure offers greater access and egress for maintenance schemes than more conventional tokamak layouts, and compatibility with advanced divertor configurations. Conversely, the design challenge is increased compared with conventional aspect ratio tokamaks:

— Optimization of bootstrap fraction to reduce the need for an auxiliary current drive—operation at high *β*_N_ and *κ*.
— New type of plasma turbulence and therefore high degree of uncertainty on the performance and controllability.
— Significantly increased control challenges (exhaust, high *β*, high self-driven current fraction)—the plasma may be more self-organizing and less susceptible to feasible actuators than some fusion concepts.
— Compact design
  - Regions unavailable for tritium breeding;
  - Less surface for handling heat and particle fluxes;
  - Less space for a central solenoid, making start-up and controlled termination more demanding.

## 3. Approach

A tokamak is a complex cobweb of interconnected systems made uncertain by unfledged understanding in the relational connections and mutual dependencies. Complex systems are characterized by emergent behaviour. This means these systems exhibit properties that would be difficult, if not impossible, to predict in advance owing to either discovery of new science or the interactions between individual components that can lead to the emergence of new behaviours or properties that are not present in the individual components. Complex nested systems are characterized by high degrees of connectivity and inevitable intrinsic frailty with greater potential for systematic collapse from triggering tipping points that cascade across systems [9]. The ITER magnet system development noted this characteristic in its journey to realization [10] and expressed alternative approaches to the traditional technology readiness level led development for fusion demonstrators [11]. The European DEMO programme has exposed the integrated nature of designing a tokamak-based power plant through its ‘Key Design Integration Issues’ [12], highlighting both the functional and spatial configuration items in tension while seeking a self-consistent design. To minimize development risk to the programme European DEMO looked to use modest extrapolations to the ITER physics and technology basis. However, several critical interrelated issues were identified at the pre-concept gate review G1 with an implicit outcome that this approach would need further consideration in the subsequent phase [13]. Two key issues stand out: the lack of a consistent plasma solution at the time and the absence of a credible remote maintenance solution for large in-vessel components. These challenges highlight that the technical and integration complexities were greater than initially assumed, particularly the oversimplified extrapolation from ITER and reliance on basic empirical scaling. The fourth dimension of integration is time spanning a spectrum from femtoseconds to decades, providing a temporal framework encompassing the dynamic phases of plasma pulse operation, including start-up, ramp-up, flat-top and ramp-down. Concurrently, knowledge and understanding will increase across the evolution of design maturation, rigorous testing, meticulous construction and the successive stages of commissioning and operation. Time becomes a critical factor in the accumulation of radiation dose, inducing consequential changes in material and component behaviours. More generally, the aging of the facilities further underscores the intricate interplay of time with the technological evolution of the STEP Prototype Powerplant (SPP). It must be acknowledged that the level of design complexity will lead to timescales that are very lengthy even if deriving a satisfactory solution is possible at all, and it is based on STEP accepting at the outset that it is only feasible to adopt an iterative evolutionary approach to the solution. Each iteration will reveal more about the interdependencies or trade-offs, and each cycle will be able to use better information and modelling. Issues are therefore uncovered and solved more rapidly this way because the effort is focused towards an end. Sufficient discovery is needed during the early stages to identify a suitable starting point or points, but then progress must be driven rapidly from those points precisely because of the interdependent nature of the problem. It is critical to establish what might work and what will not work as the final concept will inevitably be a compromise of competing factors. STEP’s design approach was simple: cadence driven through a decision architecture alongside a concept maturity (CML) [14] progression, with exploration facilitated using contrasting design families like the ARIES [15] approach, and multifactorial decision-making using measures of effectiveness (MoE) linked to the programme objectives [16] as arbitrating performance targets. Design families were evolved using a digital design workflow that connected plasma, tokamak and plant design tools into a self-consistent hierarchical functional configuration.

Achieving an integrated design hinges on adeptly balancing pivotal decisions, ensuring the overall design is both compelling and self-consistent. The exploration of the parameter space and understanding the major design family decisions provides a context that can be used to construct a concept for further development. Identifying key aspects of the programme objectives [16] can be used to steer the design direction. Metrics have been generated against these objectives: the MoE. These key plant characteristics enable the evaluation of the prospective integrated concepts against a common set of self-consistent criteria. The goal is to formulate an optimized decision set that facilitates the attainment of a balanced compromise. The MoEs considered are as follows:

— *Net power out*: Generate 100 MW_e_ net baseload power to the grid, for continuous periods (hours) of operation. Operate a plasma scenario with viable start-up and shut-down, and non-inductive current drive flat-top operating point.
— *Fuel self-sufficiency*: Demonstrate fuel self-sufficiency by breeding enough tritium to at least maintain the existing inventory. To allow for predicted losses and decay this translates to a tritium breeding ratio (TBR) of at least 1.1, supported by plant utilization needed for self-sufficiency [17].
— *Safety and environment*: Demonstrate to UK authorities (Health and Safety Executive; Environment Agency) that hazards and risks are As Low As Reasonably Practicable.
— *Maintainability*: Demonstrate a plant operational cycle, including full maintenance, that gives a pathway to a commercial powerplant (allowing for development of component lifetimes through advancements in materials).
— *Development flexibility*: This is required to allow for uncertainties in the design, allowing us to demonstrate a fusion powerplant. Subsequently, this enables operation as a fusion development facility, providing the ability to optimize operating parameters and upgrade components to further bridge the gap to a commercial powerplant.
— *Schedule*: Provide a fusion power plant demonstration as fast as reasonably practicable with operations targeted for the 2040s.
— *Cost*: Deliver SPP demonstrations and wider benefits at the lowest practicable lifecycle cost.

While fundamental performance metrics such as net power and fuel self-sufficiency can be quantified through well-defined calculations, the determination of other objectives relies heavily on the careful selection of relevant indicators aligned with the design’s fidelity. This unavoidably introduces an element of subjectivity, necessitating cautious interpretation of conclusions. The approach involves making design decisions based on the available information, with rigorous documentation of the underlying rationale. As the design evolves and comprehension deepens, a vigilant awareness of the foundational principles guiding decisions is maintained, ensuring ongoing consistency. While detailed decisions offer valuable validation, they also pose challenges that may demand adjustments or alterations during development.

## 4. The art of managing compromise

**Figure 1.**
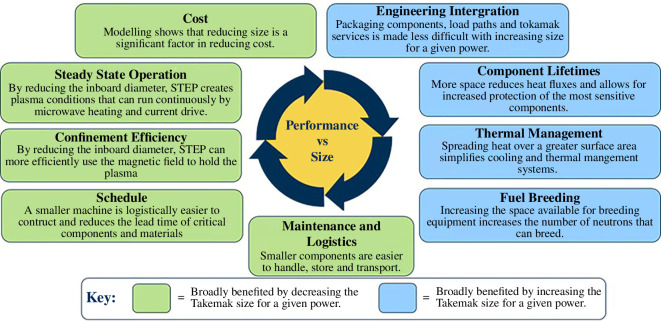
Performance versus size—the principal factors.

The trade space distils in its simplest form to be one of performance opposing size that fundamentally drives all aspects of the STEP plant design and the technical risk profile. This multifactorial challenge looks for a robust envelope with margin in the programme (whole cost and schedule), performance (power, tritium self-sufficiency, maintainability, development flexibility) and machine (proximity to technological limits and tipping points) domains. However, in the very act of the pursuit of margin, there exists a trade-off where uncertainty is often heightened, whether through larger assumptions, increased risk shifting to the future or a combination thereof. Figure 1 provides a simplified summary of the trade space.

In the early conceptual phase, a vision driven by theoretical principles laid the foundation, coordinating centralized control over strategic integration decisions and nurturing dynamic exploration within well-defined boundaries. Amid a paucity of information, essential assumptions were formulated, laying the groundwork to substantiate or retire hypotheses in the innovative design of competing solutions. Sizing has been set primarily by fusion power, operating just above the no-wall beta limit with a high bootstrap fraction for reduced recirculation power, toroidal field (*B*_T_), aspect ratio (*A*), major radius (*R*) and elongation (*κ*). The latter four components, *B*, *A*, *R*, *κ* could be said to be the major engineering parameters, largely defining the exoskeleton of the tokamak [18]. Neutral beam heating and current drive have been deselected given the hypothesis of minimizing the tokamak boundary, as have all other schemes except for microwave heating and current drive at electron cyclotron frequencies. Particularly worthy of note is the inboard build, which is the radial configuration from the centre of the machine to the inboard edge of the plasma. The overall size of the tokamak can grow significantly because of relatively small increases in the inboard build. This means that increasing the space allocation in the inboard region has the potential to fundamentally undermine the value proposition of the programme. No inboard breeding is an implicit consequence (breeding blankets are thicker than dedicated neutron shields) and the resulting increase in the technological demands on the outboard breeder blanket modules is discussed in [17]. Packaging the numerous inboard components within the initial target inboard radius of 1.6 m is technically challenging owing to the magnetic requirements for the toroidal field coils and central solenoid, while also providing enough shielding against damage from neutrons to ensure an adequate component life for a high capital cost item. This primary size and performance trade involves balancing the reduced size and, consequently, capital costs with a potentially shorter lifetime driven by heightened wall loading, thereby incurring higher operational costs. Prospecting for a balance on the holistic cost, with a focus on achieving sufficient lifetime to establish a pathway to high availability, requires a delicate compromise with other competing objectives [19]. A highly creative multi-disciplinary team was established to tackle this challenge in readiness for the review at the conclusion of this programme (Concept) phase. This will be discussed in the accompanying confinement paper [20] and later in this article with the conclusion and subsequent remedial actions. STEP aims to reduce maintenance cycles by improving resilience through seeking targeted margin gains, developing robust contingency plans, enhancing system redundancies and maximize maintenance efficiency by developing an innovative architecture that enables an alternative vertical maintenance strategy, to port-based schemes, which has several distinctive attractions described in [21]. Experience has been drawn from facilities such as JET, ITER, the European Spallation Source (ESS), sub-sea, and fission plants to develop an informed strategy. Replacing the coils requires joints in the toroidal field coils and a development and testing campaign has been established to underpin this target which is described in [21]. Material selection lies at the heart of so many of our technological choices. Lower technological magnet concepts were considered in the early trade studies using cryo-cooled aluminium with an advantage for irradiation damage recovery schemes requiring less shielding but incurring higher parasitic loads that significantly threaten the power balance—a pivotal consideration that ultimately led to the selection of high-temperature superconducting (HTS) tape as our magnet technology. STEP aims to lead the way as a pioneering fusion device, generating practical power with a target export of 100 MW of electricity (100 MW_e_) to the grid. As outlined in [22], balancing plasma performance, plasma stability and the energy required to sustain the plasma is critical to ensuring that STEP reliably produces more energy than it consumes. Fundamental to the scenario design is an efficient heating and current drive (HCD) solution that reduces the associated parasitic load. The STEP magnetic field and density have been chosen such that both electron cyclotron current drive (ECCD) and electron Bernstein current drive (EBCD) can be used in the same plasma. This mix was selected based on a series of factors including plasma HCD functionality, engineering constraints, cost impacts and RAMI [23]. Conversion efficiency to electricity is maximized by selecting high-design temperature (>600°C) primary coolants and by using a Hybrid sCO_2_ Cycle that offers greater flexibility. The higher temperature on the outboard blankets presents further constraints, as described in [24] limiting material choice given the challenging environment, process fluid compatibility, waste implications and demanding functional requirements. The restricted space within STEP means that the most efficient breeding techniques must be used to attain a sustainable fuel cycle. Once in stable operation, STEP aims to produce as much fuel as it consumes, plus a small margin to account for uncertainties, losses and to potentially start follow-on devices. This requires a multiplicity of approaches requiring the use of novel high-performance breeding materials, minimizing the amount of neutron-absorbing structural material, maximizing blanket coverage and strategic use of multipliers [17]. Harsh operating conditions degrade tokamak components, and the combined loads introduce new failure modes, meaning that they must be replaced periodically—the frequency of replacement is tightly linked to the plant availability, with different requirements for SPP and a deployed fusion plant.

## 5. Unprecedented challenge

Qualification is challenging given that the proposed operating space is a large extrapolation from current empirical experience and a full-scale integrated precursor test is not feasible almost by definition. Consequently, it is likely to be necessary to place significant reliance on *in silico* qualification to bridge this sizable gap. All models are however idealized representations and wrong to some degree, but with the prospect of discovery and new science, the confidence in those models can be eroded given the radical epistemic uncertainty [25], particularly in the plasma physics and the material science domains. JET however has successfully operated in an emergent space using margin to manage uncertainty, phased operations to manage risk and technological evolution to optimize the performance. Similar parallels can be drawn from the early stages of gas-cooled fission reactor technology, at the outset of which, minimal understanding existed regarding the implications of neutron spectra on core and vessel materials, beyond rudimentary assessments of embrittlement and hardening. Notably, no specialized nuclear engineering code was tailored for reactor pressure vessel design and verification. To surmount these challenges, reactor designers embraced a pragmatic methodology, leveraging the available knowledge while prioritizing continuous learning throughout the plant’s lifecycle. Over four decades, these reactors operated successfully, transitioning from a mode of condition monitoring to predictive performance evaluation and the demonstration of safety margins. A pivotal moment in this evolution was marked by the development of the R6 CODE and the integration of fracture mechanics into safety assessments [26]. Understanding the implications of poorer or improved performance with escape routes can be facilitated by scenario modelling on a digital shadow or virtual representation of the candidate system(s). While virtual model(s) prove valuable in comprehending the potential impact of changes in the design of SPP, it is crucial to acknowledge that their utility is constrained within a certain range of uncertainty. If the uncertainty reaches a point where it becomes challenging to handle, a flexible approach will be required. Given the significant extrapolation, and the spectre of epistemics, remaining open to a diverse array of strategies is essential to protect against unforeseen challenges. Strategies like (i) programme flexibility in expectation, (ii) engineering and proximity to limits, (iii) integration resilience supported by a system of systems consequence modelling, (iv) flexibility to allow upgrade pathways as mitigation, (v) improved knowledge through R+D and targeted start-up, and (vi) capability build of the delivery team to cope with emergent behaviour. This necessitates careful stakeholder engagement raising awareness of the risks in achieving the programme objectives but noting that experimentation is needed to arrive at viable solutions to complex system problems. That is the essence of entrepreneurship—experimental risk-bearing, leaps of faith and new levels of thinking for new challenges [9]. Our concept of operation acknowledges that our virtual representation of our physical assets will evolve concurrently with our early operational experience and increased knowledge through the plant conceptual, design optimization and operational performance phases [27]. This targeted testing and operation approach not only streamlines the development process but also allows for a more rapid iteration of design improvements, significantly reducing testing overhead and accelerating overall project timelines [24]. Plasma confinement has a significant influence on the whole-reactor design [28]; a good plasma transport regime—low enough and behaviour controllable—is needed to (i) keep the reactor core compact, (ii) minimize the current drive requirements and (iii) provide a manageable exhaust. Difficulty in obtaining saturated turbulence simulations and transport predictions in STEP-like equilibria enhances uncertainty in fusion power predictions. Making provision to be able to increase heating and current drive is a strategy to recover plasma performance based on confinement time is broadly proportional to plasma current. Flexibility to access higher current regimes would be beneficial, but it is tensioned against inefficient non-inductive current drive.

## 6. Design space prospecting

**Figure 2.**
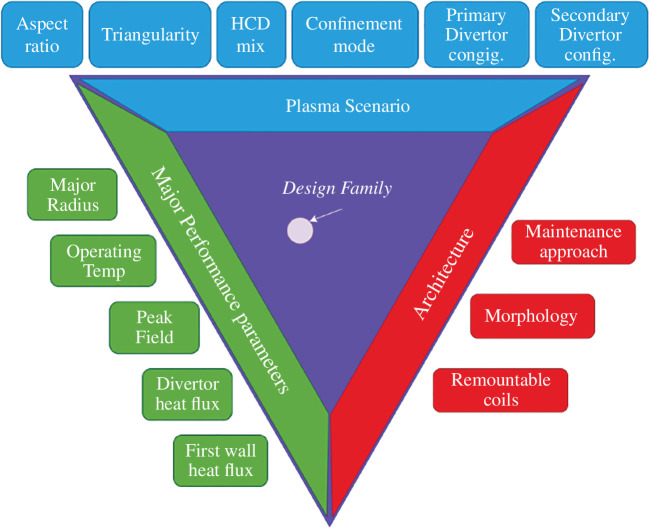
Design families.

The highly integrated nature of a fusion power plant, as illustrated in figure 1, leads to a complex design space with many options and constraints. To create a robust initial design point, the identification of technologies and an exploration of the trade space needed to be performed. To make this a more manageable problem, different concepts for the SPP were created using design families, and these concepts could be evaluated against the MoEs to measure their success. Design families represented the highest level of architectural decision making which would result in an option or technology choice, that would in turn create a constraint on the design. They often resulted in discrete choices, as opposed to an optimization of a continuous parameter. The family tree could then be used to see how the different technologies impact the design. At the highest level, decisions can be split around three families: plasma scenario, architectural and major performance parameters, as illustrated by figure 2.

The key options within the plasma scenario family are the choice of confinement mode, positive or negative triangularity, the heating and current drive methods and the divertor configuration. Negative triangularity has been shown to produce high confinement L-mode plasmas [29], an attractive option compared with more well-understood positive triangularity ELMy H-mode plasmas. I (Improved) and QH (Quiescent H) mode were also investigated as ELM-free scenarios. Neutral beam injection and microwave heating (ECCD and/or EBCD) were considered for heating and current drive. For the divertor configuration, there was a primary choice on single- or double-null. Double-null has multiple advantages such as being able to distribute heat over a larger area and channelling more of the energy to the outboard side of the tokamak where it can be more easily handled. Single-null, however, may be advantageous from a maintenance and control point of view. The secondary divertor configuration was based on the shape of the divertor, with conventional, X and Super-X considered. This is a trade-off between improved heat handling at the expense of a more complex magnetic geometry.

For the architectural decisions, the main choices were around the internal layout of the tokamak, the maintenance approach and whether to use remountable joints in the toroidal field magnets. As ARIES ST [30] asserted in a resistive coil context, remountable joints offer a radical change in maintenance by removing the constraint of port size for components. By being able to open the machine up, larger components can be removed, simplifying maintenance and accelerating timescales. This benefit is tensioned by the increased emphasis on ex-vessel contamination control and asset protection [21]. The maintenance approach reflects whether components will be removed vertically or horizontally. The internal layout looks at the use of a traditional tokamak layout of the first wall–blanket–vacuum vessel, or whether to use more radical technology like the liquid immersion blanket concept proposed for ARC [31].

The final group of decisions is the major performance parameters, which are linked to the underlying technologies required to deliver them. The choice of materials sets limits on the heat flux on the first wall and divertor. The peak field strength on the TF coil is linked to the material the coil is made of, such as cryogenic-aluminium, or low- or high-temperature superconductors. The operating temperature of the blanket heat transfer fluid, where higher gives better thermal-to-electric power conversion, is limited by the operating window of the blanket structural material.

The options selected from the design families create a set of inputs that can then be turned into a powerplant concept. This is done through a design workflow developed for STEP [32]. Initially, the systems code PROCESS [33–35] is used to create an outline of the plant, with the plasma being studied further using the fixed-boundary equilibrium code JETTO [36]. A plasma equilibrium and PF coil layout is created using the free-boundary equilibrium code FIESTA [37]. Initial designs that look promising in relation to the MoEs can be further progressed to study the inner TF coil, the TBR, the power balance and exhaust. An important principle of this design workflow is that it is based around on assumptions integration. Initial assumptions are made, and a design and level of performance are developed around those assumptions. This does not guarantee that the performance can be delivered, and extensive research and development are needed to crystallize the designs that are produced. The key is to identify the critical technologies, constraints and uncertainty that dictate the R&D plan that follows. More detail on the workflow can be found in [32].

The generation of concepts created a basis on which further analysis could be performed, and through the design families, this could identify promising branches or rule out others. For example, within the plasma families, negative triangularity was ruled out as highly elongated negative triangularity plasmas were found to be unstable in the second stability region for ballooning modes, limiting *β* [38]. Neutral beam injection (NBI) requires large access areas in the wall, reducing the breeding ratio, while also occupying large amounts of space around the tokamak. For this reason, NBI was also ruled out.

For the major performance parameters, concepts were generated using cryogenic-aluminium as the conductor.

For concepts with low toroidal field strength (approx. 2 T), taking advantage of high *β* operation, feasible concepts were found. Once the field increased beyond this, then the increase in current in the conductor led to significantly more resistive heat that increased the cryoplant demand. To overcome the new cryoplant power demand, the fusion power needs to increase to maintain the same net electrical output. This starts to spiral, as it then changes the field demand which increases the cryogenic demand more. The other impact of the increase in cryogenic demand is the increase in the size of the cryoplant. The advantage of cryogenic-aluminium is it is cheaper than superconductors, however when the additional cost of the cryoplant is included this is no longer the case for higher field devices.

In total, 66 concepts were developed to varying degrees of fidelity. Based on the exploration of concepts, EC and EBW were chosen as the heating and current drive options that gave the best current drive efficiency while minimizing the amount of space required. As the toroidal field requirements for coupling these two mechanisms to the plasma are conflicting, with EC requiring a high field and EBW low field, a toroidal field of 3.2 T was chosen to align with the overlapping region where both options work for the given plasma density. This field requirement on the geometric major radius required HTS magnets to deliver it in the limited space.

**Figure 3.**
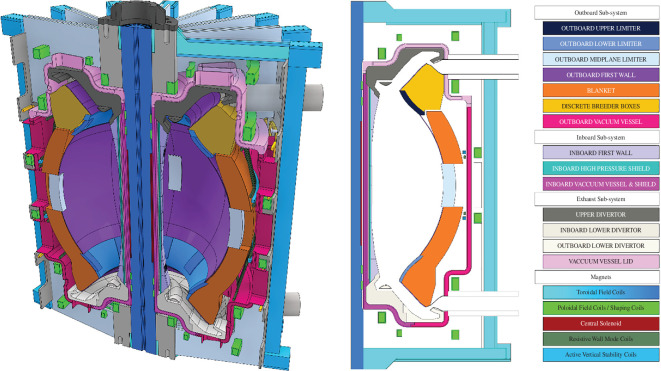
Cross-section of the spherical tokamak postulated in this special issue. The left image gives a three-dimensional cut-through of the device, while the right image shows a two-dimensional slice of half the machine.

For many tokamak designs, one of the driving constraints on size is the divertor [39]. However, for the SPP, it has been found that the minimum size of the inboard build is the driving size constraint owing to the inner space required and the use of a double-null Super-X divertor. This is not to say that the divertor is not a constraint when extrapolating to more powerful devices or for spherical tokamaks in general. When reviewing the size of all inboard components, a minimum inboard build of 1.6 m was adopted. An aspect ratio of 1.8 was chosen as the largest aspect ratio that did not require inboard breeding, giving a minimum major radius of 3.6 m. The remaining design parameters were chosen to maximize performance against the MoEs. Table 1 summarizes the key design decisions and assumptions described above, while table 2 gives the key design parameters calculated based on them. Figure 3 gives a cross-sectional view of the spherical tokamak.

**Table 1.** Key design decisions and assumptions for the current STEP Prototype Powerplant.

triangularity	positive	number of remountable TF coils	16
plasma edge	edge pedestal	remountable joints number	three per TF coil
heating and current drive mix	electron cyclotron and electron Bernstein waves	tritium breeding material	lithium
primary divertor configuration	dynamic double-null	outboard first wall, outboard limiter and blanket coolant	helium
secondary divertor configuration (inboard)	Flat-top: X-type Ramp-up: perpendicular	blanket coolant outlet temperature	600°C
secondary divertor configuration (outboard)	extended leg	centre column coolant	water
TF conductor type	HTS (REBCO)	divertor coolant	water
Tokamak morphology (radial build)	plasma–first wall–blanket–vessel	peak steady-state divertor heat flux	<20 MW/m^2^
primary maintenance access route	vertical	direct or indirect cycle	indirect

**Table 2.** Key design parameters for the current STEP Prototype Powerplant, calculated on decisions and assumptions basis in table 1.

fusion power	1600–1800 MW	magnetic field	3.2 T
net electric power	100–200 MW_e_	elongation	2.93
major radius	3.6 m	triangularity	0.5
inboard radius	1.6 m	plasma current	20–25 MA
aspect ratio	1.8	tritium breeding ratio	1.17

## 7. Whole-plant performance

The SPP design is shaped by decisions building on initial design choices. While these decisions can be made independently, it is crucial to ensure they integrate to collectively achieve the project’s goals (MoEs). The design focuses on key pillars, aiming for net power and fuel self-sufficiency. A major challenge is the uncertainty in plasma design regarding performance and control, given limited data and scalability concerns, posing a significant risk. To mitigate this, STEP plans to engineer a flexible design that accommodates various plasma scenarios, allowing for exploration and improvement based on initial test results. The identified parameters from this exploration guide the foundational requirements for concept development.

The breeding blanket is pivotal in achieving fuel self-sufficiency and net power within the STEP powerplant. It serves as the nexus connecting the tokamak, fuel cycle and power and cooling systems. This pivotal component presents formidable challenges, accentuated by the reduced volume of the compact design approach, necessitating strategic compromises. The primary focus centres on achieving fuel self-sufficiency, quantified by the TBR—the ratio of tritium bred per tritium consumed. Maintaining a TBR of at least 1.1, accounting for operational losses, is a guiding principle.

To optimize compactness, spatial constraints in the inboard region lead to the exclusion of breeding function in this region. While breeding provides some shielding, its inefficiency in space utilization compels forgoing inboard breeding. The targeted TBR of 1.1 guides the breeding technology choice, seeking a robust solution amid evolving design uncertainties. Extensive research [17] highlights the superiority of liquid lithium with 35–45% enrichment (^6^Li) in maximizing TBR for our reduced size design.

An intriguing possibility arises from integrating breeding and cooling functions using lithium as a breeder. However, the formidable magnetohydrodynamic loads within a high magnetic field render this solution impractical [17]. Consequently, a separate helium coolant was selected, aligning with lithium compatibility. Coolant selection requires a delicate balance of critical factors, including gross thermal efficiency, pumping power, material suitability, spatial efficiency, safety, plant availability, neutron shielding, acquisition and development costs.

The blankets and outboard first wall are the primary heat sources within the tokamak, generating 65% of the total available heat. Optimizing their design is crucial for effective heat management in the SPP. To achieve thermal conversion efficiency, we maintain the outlet temperature at a critical 600°C. This temperature, along with breeder selection, dictates the structural material for the blanket: vanadium. Vanadium elegantly preserves key material characteristics at elevated temperatures and strategically counters normal tritium absorption tendencies, thanks to the presence of the lithium breeder. Importantly, vanadium avoids compatibility issues with lithium.

While other components require cooling, their heat production occurs at lower temperatures owing to managing high heat fluxes, especially from divertors and limiters [40]. Our supercritical CO_2_ cycle optimally configures heat inputs, with only 2% deemed unusable [22]. Decisions on coolant selection are intricately tied to system requirements. Inboard shielding, critical for maximizing capability, uses water. However, we contemplate heavy water (D_2_O) owing to its reduced neutron absorption properties yielding a higher TBR. For divertors, D_2_O is chosen for superior heat transfer capabilities, balancing heat transfer rates with pumping power considerations—a factor impacting net power delivery.

Finally, limiters employ helium cooling, a deliberate choice owing to their resilience against extreme loads, particularly during transient events. In the unfortunate event of a Loss Of Coolant Accident, helium minimally impacts other systems, balancing consequential and recovery impact. This nuanced coolant selection aligns with our overarching goal: designing a fusion powerplant that balances essential safety, performance and availability requirements.

Evaluating our performance metrics yields a positive outlook: 149 MW_e_ net power predicted, alongside a TBR of 1.17—a proxy for fuel self-sufficiency. Our design offers flexibility by varying the HCD between ECCD and EBW, with the capability to exchange all in-vessel components.

However, ensuring availability remains a significant challenge. Long maintenance cycles are necessary for HTS coil and structure warm-up and cooldown, as well as vacuum pumping. Unplanned events pose risks, especially during early operational phases when plasma control is under development. We must harness limiters as sacrificial technology for design protection and develop maintenance techniques for issue detection. Beneath the performance predictions lies uncertainty. Many design aspects rely on unvalidated technologies within the operational envelope. Before plasma performance can be validated through machine operation, theory and simulation play a crucial role in reducing uncertainty by allowing controlled exploration and understanding of complex systems therein. Sizing the machine hinges on novel partially insulated HTS TF limbs. Achieving fuel self-sufficiency depends on unproven tritium designs and suitable extraction and processing technologies.

Navigating the high-risk landscape—encompassing both final performance and plant realization—presents a stark challenge for the STEP programme. However, through meticulous analysis, the key sources of greatest uncertainty and sensitivities therein can be pinpointed. Armed with this understanding, STEP drives testing and development of critical technologies.

While residual uncertainties will persist, particularly regarding plasma performance and control, the programme arms itself with a diverse array of strategies to cope and adapt. Simultaneously, it carries out relentless mitigation of technical risks through rigorous testing and demonstrations of critical design elements.

## 8. Challenges in control

When considering the time domain, one of the key challenges that goes beyond the basic design is the ability to control the array of complex systems that compose the powerplant. SPP will require a multi-disciplinary effort that integrates methodologies from various industries, including automotive, aerospace, data management and scientific facilities. Achieving fusion’s ambitions demands collaboration among experts to adapt existing approaches and develop innovative technologies.

Controlling plasmas in a tokamak mirrors the challenges of rocket flight, demanding high-precision, real-time control for rapid responses to ‘off-normal’ events that could risk system damage [41]. However, the analogy falls short as fusion control involves integrated management of numerous components in an exceptionally challenging environment, coupled with a less comprehensive understanding of the fundamental physics. This complexity necessitates the management of emergent behaviour as it arises during operation, acknowledging the potential breakdown of traditional causality frameworks inherent in more well-understood systems.

Fusion control systems face unique environmental challenges owing to radiation, electromagnetic interference (EMI) and extreme temperatures. These factors drive the need for innovative solutions in measurement, actuation, data transmission and signal processing. Gamma radiation post-plasma operations requires sophisticated remote maintenance (RM) equipment. During plasma operation, both neutron and gamma radiation necessitate new methods for monitoring and control, mitigating noise and addressing temperature extremes and magnetic field effects. Advances in radiation-tolerant materials and remote measurement systems are also underway.

Operating a fusion powerplant demands novel, high-performance control schemes for complex, integrated areas. Controllability extends beyond mere system installation; it requires precise measurements, responsive actuators and well-behaved plasma. Plasma control, in particular, plays a crucial role in managing various tightly coupled plant systems. Additional challenges involve managing the fuel cycle, incorporating intricate tritium monitoring, extraction and storage technologies while balancing particle/gas versus heat in the exhaust and fuel injection. Ensuring compliance with regulatory and commercial requirements necessitates strict control of tritium inventory within the system [17]. Moreover, the power infrastructure must seamlessly integrate and control complex systems, addressing the significant, fluctuating energy inputs and outputs of the tokamak cycle while aligning with National Grid constraints, using on-site thermal and electrical storage systems [22].

For intricate control of tokamak-based powerplants, STEP will employ models of the plant, plasma and operational environments to identify and optimize control strategies. These models integrate existing physics knowledge with emerging experimental data, enabling the exploration and refinement of various control strategies. This approach facilitates expeditious design development, minimizing the time-consuming and costly aspects of incremental system development and reducing risks associated with high-energy systems like plasma. These models also support the development of STEP’s control algorithms through the use of the physics models to generate control-oriented equivalents capable of running in real-time, such as through training neural networks using reinforcement machine learning (ML) techniques. In applying such techniques consideration is being given to the level of integrity associated with the physics model data and uncertainty associated with the resulting control algorithms [42].

As part of concept development, and in alignment with the UK Government’s green paper on a regulatory framework for fusion [43], instrumented safety systems for STEP shall be developed in line with a Process Industry functional safety standard [44]. As such, Safety Integrity Levels (SIL) shall be used to define integrity associated with safety functions and systems. The SIL approach shall also be applied to asset protection with a direct translation of SIL to Protection Integrity Level, in relation to associated requirements, and allocation based on risk tolerance as defined by STEP.

Use of complex control systems for asset protection is unavoidable as one of the key challenges in protecting our fusion plant is preventing the high-energy plasma from damaging the tokamak. Achieving such protection requires the development of systems capable of both analysing plasma for the onset of events and actuating plasma to eliminate or mitigate events, both of which require complex control strategies and technologies. For successful delivery of these systems, STEP is developing novel strategies and technologies for the protection of the tokamak. Complex ‘safe-landing’ strategies to prevent damage to the tokamak machine and its sub-systems during off-normal plasma events are discussed in [41].

In a fusion plant, the interdependence of various systems means that events in one part can significantly impact others. Protection in STEP is addressed through two tiers: the first ensures individual system safety and asset protection, preventing harm and damage locally; the second operates at the whole-plant level, coordinating site-wide responses to maximize safety and minimize overall damage and costs. Whole-plant systems can override local actions when their consequences are outweighed by the broader site impact. Critical functions shall be designed such that they are single-failure tolerant, ensuring graceful degradation of functional performance rather than a ‘cliff-edge’ response. This shall be achieved through the application of redundancy and diversity at both system and functional levels. STEP shall also implement combinatorial control techniques in relation to critical and complex control challenges, such as plasma control. Within complex, highly integrated processes a change of one parameter, such as plasma current or density, is coupled to every other process parameter to varying degrees. This coupling of process parameters provides the opportunity to infer or affect plasma parameters indirectly through knowledge of the relationship between separate but related parameters. This can be exploited to allow selection of a greater range of measurement and actuation technologies as well as the mitigation of uncertainty in physics and plant performance by combining multiple measurement or control functions together to reduce error and provide self-verification.

## 9. Concept of operation and phasing

The operating point of STEP will be significantly different from today’s tokamaks, extrapolating into a very challenging and hitherto unprecedented operating space. In the past, machines like JET have also grappled with similar challenges, successfully navigating commissioning processes that deviated significantly from existing operating points. What is the critical pathway to reaching the desired outcome remains a topic of much debate. Maximizing time in non-active operations is often assumed to be impractical given the cost of installed power to mimic alpha heating, time spent deferring, and it is not fully representative of deuterium–tritium (D–T) phase intricacies particularly the current profile dynamics, and roles of fusion alphas. Valuable insights can be gleaned from an extended hydrogen phase, with repairs being notably easier. However, determining the criteria for success or failure in terms of confidence that the D–T phase will yield the required plasma performance and maintain controllability, thereby avoiding intervention-triggering events, remains ambiguous. Time in integrated commissioning is useful to shakedown the system of systems behaviour, improve model confidence and build operator capability ahead of active operations. But like other analogous programmes, the primary objective is to seize expedient opportunities wherever feasible [45].

Operations will insightfully develop to build operational and scientific expertise in machine operations and hazard management across the entire plant. To accomplish this, the SPP operations are separated into three key phases: plasma development and validation, high-power plasma validation and initial tritium breeding and power plant demonstration operations.

The initial phase focuses on plasma development and gaining foundational knowledge of the machine and early scenarios, including operating with hydrogen and deuterium plasmas. The main goal of this phase is to develop robust plasma operating scenarios, while gradually increasing plasma heating power and plasma pulse duration, which will subsequently be used during D–T plasma operations. This phase will use comprehensive engineering and scientific diagnostics to validate tokamak design and to measure plasma parameters. Commissioning of various machine protection systems, in particular disruption mitigation, is another important task that will be completed during this operational period. This phase will also demonstrate remote handling operations prior to significant machine activation, and tritium accountancy techniques prior to tritium usage in the tokamak.

The second phase, high-power plasma validation and initial tritium breeding, will consist of high-power D–T plasma operation. This phase will focus on building from the plasma operating scenarios developed in phase 1. This operation will be carried out with a reduced set of engineering and scientific diagnostics owing to the increased radiation field within the tokamak.

The third phase is the power plant operations demonstration. During this phase, STEP will operate as a power plant, which includes exporting electricity to the National Grid. This phase will also include optimization of operational and maintenance procedures and the demonstration of commercially relevant objectives.

Following the three phases of STEP operation, the plant will be available for further exploitation. This period is reserved for future developments which will support the development of the commercial fusion industry. At this point in time, there is little detail available, however, it is expected that this may include testing of novel materials, breeding techniques or optimizing system efficiencies.

## 10. Conclusions

STEP has pushed the compact hypothesis hard towards a threshold where cost advantages of size have been overtaken by the stronger relationship with complexity. The programme has embraced a heightened risk tolerance within the framework of a set-based concurrent engineering design methodology [46]. The evolution of design fidelity proceeded along a structured decision tree, strategically directing endeavours, facilitating accelerated knowledge acquisition through rapid adaptation within the constraints, exorcising the sensitivities and extracting valuable insights from encountered setbacks. The shortened timescales have been realized through a model-based workflow that enabled aggressive design experimentation and targeted testing programmes. However, this is a significant extrapolation into a new space given that a full-scale integrated precursor test is not feasible, almost by definition. Theory acts as a visionary guide [47,48] to traverse this liminal space supported by system scenario modelling to test the asserted hypothesis and solution. Albeit that discovery often re-writes the rules, and this approach has worked well in building the required capability to work confidently in this emergent space. Much like a virus or pathogen that continually mutates, the pursuit of higher design fidelity introduces novel complexities. These design intricacies act as dynamic adversaries, demanding constant adaptation and resilience. In this context, the strengthened capabilities of the team serve as the equivalent antibodies—nimble, responsive and adept at countering the ever-evolving challenges posed by the intricate design landscape. Inevitably conflicts arise that prevent the decision pathway from being straightforward and balances of pros and cons incur costs for the sake of a greater good. Based on the explored point, the programme has chosen to relax the constraints by investigating a larger radial build, reduced elongation and increased aspect ratio. These adjustments are driven primarily by the need to improve component lifetimes, reduce spatial congestion and manage system complexity. This is still a reduced size compared with conventional tokamak geometries but importantly looks to balance the risk, improve the deliverability and robustness of the design solution. The fourth dimension of integration, time, only reinforces the constant change that the team must remain vigilant and responsive to.

As we approach the dawn of electricity-producing fusion at the West Burton site, let us reflect on the journey from JET’s first pulse in 1983 to this pivotal moment. In the spirit of the Wright brothers’ Kitty Hawk moment in 1903, and the monumental moon landing in 1969 just 66 years later, we stand in the vanguard of a transformative breakthrough. The fusion powerplant is not just a technological marvel; it signifies our collective capacity for creativity, innovation and determination.

## Data Availability

This article has no additional data.
